# Frontline aspiration versus stent retriever thrombectomy for M2 occlusions: Insights from the STAR registry

**DOI:** 10.1093/esj/23969873251381924

**Published:** 2026-01-01

**Authors:** Michael Gaub, Rahim Abo Kasem, Ilko Maier, Ansaar Rai, Pascal Jabbour, Joon-Tae Kim, Brian Howard, Ali Alawieh, Stacey Quintero Wolfe, Robert M Starke, Marios-Nikos Psychogios, Amir Shaban, Nitin Goyal, Justin Dye, Ali Alaraj, Mohamad Ezzeldin, Shinichi Yoshimura, David Fiorella, Omar Tanweer, Daniele G Romano, Pedro Navia, Hugo Cuellar, Isabel Fragata, Adam Polifka, Joshua Osbun, Fazeel Siddiqui, Mark Moss, Kaustubh Limaye, Maxim Mokin, Charles Matouk, Min S Park, Waleed Brinjikji, Ergun Daglioglu, Richard Williamson, David J Altschul, Christopher S Ogilvy, Roberto Crosa, Michael R Levitt, Benjamin Gory, Alexandra Paul, Peter Kan, Walter Casagrande, Shakeel Chowdhry, Michael F Stiefel, Ramesh Grandhi, Alejandro Spiotta, Justin Mascitelli

**Affiliations:** Department of Neurosurgery, University of Texas Health Science Center at San Antonio, San Antonio, TX, USA; Department of Neurosurgery, Division of Neuroendovascular Surgery, Medical University of South Carolina, Charleston, SC, USA; Department of Neurology, University Medicine Goettingen, Goettingen, Germany; Department of Neuroradiology, West Virginia University, Morgantown, WV, USA; Department of Neurological Surgery, Thomas Jefferson University, Philadelphia, PA, USA; Department of Neurology, Chonnam National University Hospital, Gwangju, Republic of Korea; Department of Neurosurgery, Emory University School of Medicine, Atlanta, GA, USA; Department of Radiology and Imaging Sciences, Emory University School of Medicine, Atlanta, GA, USA; Department of Neurosurgery, Emory University School of Medicine, Atlanta, GA, USA; Department of Radiology and Imaging Sciences, Emory University School of Medicine, Atlanta, GA, USA; Department of Neurosurgery, Wake Forest School of Medicine, Winston Salem, NC, USA; Department of Neurological Surgery, University of Miami Miller School of Medicine, Miami, FL, USA; Department of Neuroradiology, Clinic of Radiology and Nuclear Medicine, University Hospital Basel, Basel, Switzerland; Department of Neurology, University of Iowa Roy J and Lucille A Carver College of Medicine, Iowa City, IA, USA; Department of Neurosurgery, Semmes-Murphey Neurologic and Spine Institute, Memphis, TN, USA; Department of Neurosurgery, Loma Linda University Health, Loma Linda, CA, USA; Department of Neurosurgery, University of Illinois at Chicago, Chicago, IL, USA; Department of Clinical Sciences, College of Medicine, University of Houston, Houston, TX, USA; Department of Neurosurgery, Hyogo College of Medicine, Nishinomiya, Japan; Department of Neurosurgery, The State University of New York at Stony Brook (SUNY SB), New York, NY, USA; Department of Neurosurgery, Baylor College of Medicine, Houston, TX, USA; Department of Neuroradiology, University Hospital 'San Giovanni di Dio e Ruggi d’Aragona', Salerno, Italy; Department of Interventional and Diagnostic Neuroradiology, Hospital Universitario La Paz, Madrid, Spain; Department of Neurosurgery, Louisiana State University Health Sciences Center (LSUHSC), Shreveport, LA, USA; Department of Neuroradiology, Centro Hospitalar de Lisboa Central, Lisbon, Portugal; Department of Neurosurgery, University of Florida, Gainesville, FL, USA; Department of Neurosurgery, Washington University in Saint Louis School of Medicine, Saint Louis, MO, USA; Department of Neurology, University of Michigan Health-West, Wyoming, MI, USA; Department of Interventional Neuroradiology, Washington Regional Medical Center, Fayetteville, AR, USA; Department of Neurology, Indiana University, Bloomington, IN, USA; Department of Neurosurgery, University of South Florida College of Medicine, Tampa, FL, USA; Department of Neurosurgery, Yale University, New Haven, CT, USA; Department of Neurosurgery, University of Virginia, Charlottesville, VA, USA; Department of Neuroradiology, Mayo Clinic Minnesota, Rochester, MN, USA; Department of Neurosurgery, Ankara Bilkent City Hospital, Ankara, Turkey; Department of Neurology, Allegheny Health Network, Pittsburgh, PA, USA; Department of Neurosurgery, Montefiore Medical Center, Bronx, NY, USA; Neurosurgical Service, Beth Israel Deaconess Medical Center (BIDMC), Boston, MA, USA; Department of Endovascular Neurosurgery, Médica Uruguaya, Montevideo, Uruguay; Department of Neurological Surgery, University of Washington School of Medicine, Seattle, WA, USA; Department of Diagnostic and Interventional Neuroradiology, CHRU Nancy, Nancy, France; Department of Neurosurgery, Albany Medical College, Albany, NY, USA; Department of Neurosurgery, The University of Texas Medical Branch at Galveston, Galveston, TX, USA; Department of Neurosurgery, Hospital Juan A. Fernandez, Buenos Aires, Argentina; Department of Neurosurgery, NorthShore University HealthSystem, Evanston, IL, USA; Department of Vascular Neurosurgery, Piedmont Healthcare Inc, Atlanta, GA, USA; Department of Neurosurgery, University of Utah, Salt Lake City, UT, USA; Department of Neurosurgery, Division of Neuroendovascular Surgery, Medical University of South Carolina, Charleston, SC, USA; Department of Neurosurgery, University of Texas Health Science Center at San Antonio, San Antonio, TX, USA

**Keywords:** Mechanical thrombectomy, middle cerebral artery, aspiration, stent retrievers

## Abstract

**Background:**

Recent trials have furthered uncertainty regarding the endovascular benefit for medium vessel occlusions (MeVO). Stent retrievers (SR) were employed in the first attempt in most interventional arm participants. We sought to compare outcomes in acute MCA M2 occlusions between frontline aspiration and SR, and to delineate procedural and anatomical covariates associated with differential treatment effect.

**Methods:**

Retrospective analysis of a multicenter stroke thrombectomy cohort identified cases of MT for M2 occlusions. Unmatched and propensity score-matched (PSM) cohorts were generated comparing frontline aspiration to standalone and combined SR. The primary outcome was functional independence (mRS 0–2) at 90 days. Recanalization, symptomatic intracranial hemorrhage (sICH), mortality, and the effect of M2 laterality, division occlusion and procedure time were assessed.

**Results:**

About 1734 patients with M2 occlusions underwent either frontline aspiration (*n* = 711) or SR/combined (*n* = 958) thrombectomy between 2013 and 2024. PSM analysis favored aspiration for functional independence (49.9% vs 44.0%, OR 1.27 (1.03–1.57)), complete recanalization (61.2% vs 48.7%, OR 1.66 (1.34–2.05)), complete first pass effect (35.0% vs 27.6%, OR 1.42 (1.13–1.78)), and sICH (3.5% vs 6.2%, OR 0.55 (0.33–0.91)), with no difference in mortality. Frontline aspiration had significantly shorter procedural times (median 28 [IQR 15–49.5] vs 51 [IQR 35–78] minutes; *p* < 0.001). For every minute increase in procedure time, the probability of functional independence decreased significantly (*p* < 0.001) less with frontline aspiration (0.35%) compared to SR/combined (1.61%).

**Conclusion:**

Frontline aspiration for M2 occlusions resulted in better clinical and angiographic outcomes compared to SRs. Future trials for MeVO with a focus on contact aspiration thrombectomy may succeed where recent trials have failed.

## Graphical Abstract

**Graphical Abstract:**
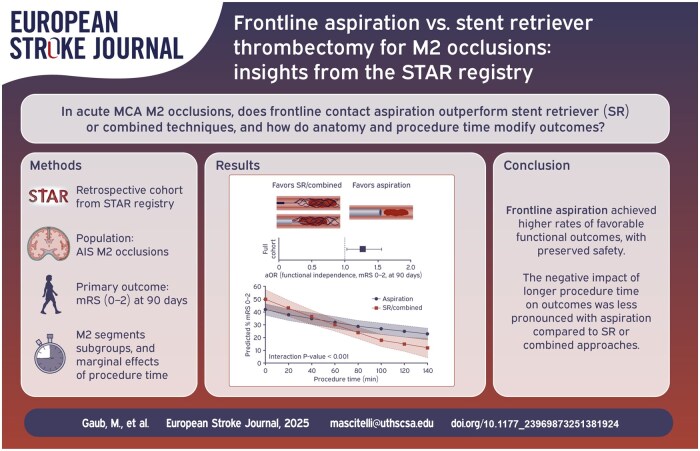

## Introduction

Seminal trials from the last decade establishing the superiority of mechanical thrombectomy (MT) for large vessel occlusions spurred an endovascular revolution in the management of acute ischemic stroke (AIS).^1–7^ In two new clinical trials, ESCAPE-MeVO^8^ and DISTAL,^9^ thrombectomy-based endovascular therapies for AIS failed to outperform best medical therapy for medium vessel occlusions (MeVO). These latest trials have received criticism for inconsistent definitions of MeVO, including heterogeneous inclusion criteria for MCA M2-segment occlusions, the heavy reliance on stent retrievers (SRs), and longer processing (picture-to-puncture) and reperfusion (puncture-to-perfusion) times compared to previous LVO trials.

There is now greater uncertainty on how best to handle MeVO. Prior non-randomized studies showed benefit of MT for MeVO, particularly those confined to proximal and dominant M2 segments.^10–13^ Meanwhile, it is impossible to deny the persuasive findings from the recent randomized clinical trials on the subject. The results of the MeVO MT trials will only further potentiate clinical equipoise for a stroke entity that one-third of neurointerventionalists already refrain from treating in the current endovascular landscape.^14^

In seeking to address this clinical issue, we asked whether a different frontline thrombectomy technique, namely a direct aspiration first pass technique (ADAPT) using contact aspiration (CA), might have resulted in different outcomes.^15^ ESCAPE-MeVO and DISTAL relied heavily on first-attempt stent retriever (SR) alone and combined approaches,^16^ with only a small minority of patients receiving frontline aspiration (0% and 15.7%, respectively). Therefore, we revisited this question within the STAR registry, which reflects real-world practice to date, by comparing acute M2 occlusions treated with frontline aspiration versus stent retriever/combined techniques. In parallel, we examined anatomical and procedural effect modifiers to better inform future trial design and individualized device selection.

## Methods

### Study population

This is a retrospective cohort analysis of a prospectively collected database from the STAR registry,^17^ a prospectively maintained, international, multicenter database with data contributed by 35 participating centers. The study was approved by the institutional review board in each contributing center. Patient-informed consent was waived owing to the anonymous nature of the investigation. The study followed the Strengthening the Reporting of Observational Studies in Epidemiology (STROBE) reporting guidelines.^18^

Consecutive patients were included from January 2013 to June 2024 if they had AIS due to MCA M2-segment occlusion and underwent MT. M2 occlusions were categorized based on **IMS III anatomical criteria**.^19^ Per these criteria, M2 occlusions are defined as MCA occlusions distal to the MCA lenticulostriates and that demonstrate antegrade filling of at least one classic M2 branch (post-bifurcation frontal or temporal divisions, pre-bifurcation posterior temporal or holotemporal branches), indicating partially preserved MCA cortical distribution perfusion. This is in contrast to M1 occlusions that are either proximal to the lenticulostriates or show absence of distal flow to the entire MCA cortical distribution. Furthermore, occlusions were classified as **trunk or division occlusions** based on occlusion position relative to the bifurcation, where trunk occlusions show distal MCA temporal distribution filling through pre-bifurcation posterior temporal or holotemporal branches but without filling of classic post-bifurcation MCA divisions.^19^ M2 occlusions and segment involvement were identified locally at participating sites; central adjudication was not performed. The decision to perform MT and the treatment technique selected were at the discretion of the proceduralist.

**Figure 1.**
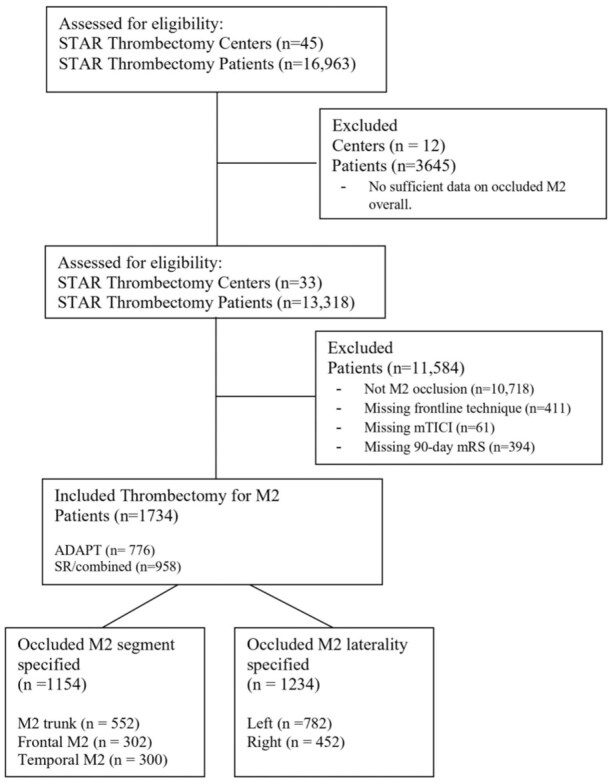
Flow chart for cohort selection criteria. Inclusion criteria include M2 occlusion treated with mechanical thrombectomy. Registry entries with missing M2 occlusion data were excluded from the analysis, including missing frontline technique (*n* = 411), missing reperfusion (*n* = 61), and functional (*n* = 394) outcomes.

Patients with missing thrombectomy technique data or clinical outcomes were excluded. A flow chart with the full selection criteria is shown in Figure 1. Each center independently collected basic demographics, medical history, comorbidities, baseline functional status using the modified Rankin scale (mRS), details of presentation including the National Institutes of Health Stroke Scale (NIHSS) score and Alberta Stroke Program Early CT Score (ASPECTS), details of the MT including location and technique, clinical outcomes, and complications. Patients were categorized into subgroups according to M2 laterality (left vs right), M2 division (frontal vs temporal), and thrombectomy technique (frontline aspiration vs stent retriever alone or combined).

### Outcome measures

The primary outcome was the rate of functional independence (mRS 0–2) at 90-day follow-up. Functional independence (mRS 0–2) was chosen over the more selective excellent functional outcome (mRS 0–1) as M2 occlusions primarily involve larger volumes of MCA cortical territories, and this metric has been used in previous M2 MT analyses.^10^ Secondary outcomes included successful recanalization, defined as a post-procedure mTICI scale of ⩾2b; complete recanalization, defined as a post-procedure mTICI scale of ⩾2c; modified first pass effect, defined as a post-procedure mTICI scale of ⩾2b with one MT attempt; complete first pass effect, defined as a post-procedure mTICI scale of ⩾2c with one MT attempt ^20^; rate of excellent functional outcome (mRS 0–1) at 90-day follow-up; NIHSS score at discharge; 90-day mortality; distal embolization; any intracranial hemorrhage; and symptomatic intracranial hemorrhage (sICH). sICH was defined as any ICH with concurrent documentation of clinical deterioration according to any one of the following criteria: (1) an increase in the NIHSS score of 4 or more points, (2) an increase in the NIHSS score of two or more points in one category, or (3) need for intubation, hemicraniectomy, external ventricular drain placement, or other major medical or surgical intervention.^21,22^ Another outcome representing the interaction between technical success and functional outcomes was futile recanalization, defined as a 90-day mRS score of 3 or above after a post-procedure mTICI scale of ⩾2b.

### Statistical analysis

All statistical analyses were conducted using R (version 4.4.3; RStudio, Boston, MA, USA). The Kolmogorov-Smirnov test was used to assess the distribution of data. Normally distributed data are presented as mean ± standard deviation (SD), skewed data as median (interquartile range, IQR), and categorical variables as frequencies and percentages. Group comparisons of characteristics were performed using the Wilcoxon rank-sum (Mann-Whitney) test for continuous variables and the χ2 test for categorical variables, as appropriate.

Multiple imputation was used to handle missing data for baseline characteristics (e.g. race, onset-to-arterial puncture time, gender, and comorbidities) to avoid associated biases. Rubin’s rule was applied to combine estimates across the multiple imputed datasets, providing adjusted coefficients for the regression models. A total of 10 imputations were performed for each model.

Propensity score matching (PSM) was performed to balance the baseline characteristics (demographics, comorbidities, and admission variables) between patients who received frontline aspiration as the frontline MT technique and those who received stent retrievers. The matching variables included sex, age, race, comorbidities, pre-stroke mRS, smoking status, intravenous thrombolysis, and use of balloon guide catheter. A 1:1 matching ratio was used, based on the largest sample size that still allowed for an appropriate balance of confounding variables. Matching was conducted using the “MatchIt” package in R,^23^ employing an “optimal” matching method that pairs patients based on their propensity scores to minimize the overall distance between the two cohorts.^24^ The balance of covariates was assessed using univariate analysis and standardized mean difference (SMD) calculations in the matched cohorts.^25^ A two-tailed *p*-value < 0.05 was considered statistically significant.

To examine differences in study outcomes across subgroups based on procedural factors, occluded M2 laterality and division, multivariate analysis was conducted using logistic regression models. The centers where participants were enrolled were treated as random effects in the regression models. All regression analyses employed a Backward-Conditional stepwise selection algorithm in SPSS to minimize bias in variable selection. To assess the impact of procedure time on the 90-day good outcome rate (primary outcome), we calculated marginal effects across different frontline techniques, considering effect modifiers and patient subpopulations.^26^ Logistic regression models were used for this analysis, ensuring that the same variables were included for all subgroups to allow for valid comparisons. Marginal effects were computed using the “ggeffects” package in R, and linear best-fit models were employed to examine the relationship between procedure duration and outcome. The goodness of fit was assessed using Pearson’s *R*^2^, which was above 0.95. The slopes of the best-fit models were calculated and compared between groups, where appropriate, using *z*-scores.

## Results

### Baseline characteristics

From January 2013 to June 2024, 2600 patients treated with MT for AIS due to MCA M2-segment occlusions were identified. A total of 866 patients had missing procedural data (frontline technique, *n* = 411) or clinical outcomes and were excluded from the cohort, resulting in a total of 1734 patients included for analysis (Figure 1). Baseline characteristics (Table 1) for the entire cohort were equally distributed by gender (female 51.1%), and the majority were white race/ethnicity (67.1%, c.f. black 16.3%, Hispanic 6.5%, and other 10.1%). The average age was 70.6 years (SD 14.0 years). Medical comorbidities included hypertension (74.1%), diabetes mellitus (31.0%), atrial fibrillation (38.8%), hyperlipidemia (46.4%), congestive heart failure (14.2%), and prior stroke (16.3%). Most were non-smokers (73.2%) while a minority were current (13.1%) or former smokers (13.7%). There was a high level of baseline functionality with a median pre-stroke mRS 0 (IQR 0–1).

**Table 1.** Baseline demographics and procedural characteristics.

Baseline characteristics	Overall (*n* = 1734)	Frontline aspiration* (*n* = 776)	SR/combined* (*n* = 958)	*p*-value*
Sex, female *n* (%)	886 (51.1)	376 (48.5)	510 (53.2)	0.048
Age, year mean (SD)	70.6 (14.0)	68.8 (14.4)	72.0 (13.5)	<0.001
Race or ethnicity, *n* (%)				<0.001
White	1164 (67.1)	533 (68.7)	631 (65.9)	
Black	282 (16.3)	144 (18.6)	138 (14.4)	
Hispanic	113 (6.5)	45 (5.8)	68 (7.1)	
Other	175 (10.1)	54 (7.0)	121 (12.6)	
Hypertension, *n* (%)	1285 (74.1)	570 (73.5)	715 (74.6)	0.58
Diabetes, *n* (%)	537 (31.0)	243 (31.3)	294 (30.7)	0.79
Atrial fibrillation, *n* (%)	673 (38.8)	273 (35.2)	400 (41.8)	0.006
Hyperlipidemia, *n* (%)	804 (46.4)	378 (48.7)	426 (44.5)	0.081
Congestive heart failure, *n* (%)	246 (14.2)	132 (17.0)	114 (11.9)	0.003
Prior stroke, *n* (%)	283 (16.3)	143 (18.4)	140 (14.6)	0.036
Pre-stroke mRS, median [IQR]	0 [0–1]	0 [0–1]	0 [0–1]	0.55
Smoking, *n* (%)				<0.001
Nonsmoker	1270 (73.2)	507 (65.3)	763 (79.6)	
Prior smoker	237 (13.7)	138 (17.8)	99 (10.3)	
Current smoker	227 (13.1)	131 (16.9)	96 (10.0)	
Admission NIHSS, median [IQR]	13 [7–18]	12 [7–18]	13 [7–18]	0.265
ASPECTs, median [IQR]	8 [8–10]	8 [8–10]	8 [8–10]	0.588
Intravenous thrombolysis, *n* (%)	666 (38.4)	264 (34.0)	402 (42.0)	<0.001
Intraarterial thrombolysis, *n* (%)	135 (7.8)	70 (9.0)	65 (6.8)	0.09
Angioplasty, *n* (%)	50 (2.9)	23 (3.4)	27 (4.5)	0.316
Intracranial stent, *n* (%)	51 (2.9)	19 (2.7)	32 (4.0)	0.201
Balloon guide catheter, *n* (%)	293 (16.9)	112 (14.4)	181 (18.9)	0.014
Procedure time, min, median [IQR]	40 [22–64]	28 [16–49]	54 [36–80]	<0.0010
Number of attempts, median [IQR]	2 [1–3]	2 [1–3]	2 [1–2]	0.273
M2 segment defined, *n* (%)	*n* = 1154	*n* = 581	*n* = 573	0.012
M2 trunk	552 (47.8)	303 (52.2)	249 (43.5)	
Frontal division	302 (26.2)	137 (23.6)	165 (28.8)	
Temporal division	300 (26.0)	141 (24.3)	159 (27.7)	
Laterality, *n* (%)	***n* = 1234**	***n* = 641**	***n* = 593**	0.008
Left	782 (63.4)	429 (66.9)	353 (59.5)	
Right	452 (36.6)	212 (33.1)	240 (40.5)	

Overall and unmatched frontline thrombectomy cohorts.

^*^ *p*-values for comparison of frontline aspiration and SR/combined frontline thrombectomy techniques.

Presentation data showed moderate stroke severity with a median admission NIHSS score of 13 (IQR 7–18) and median ASPECTS of eight (IQR 8–10). A minority were administered intravenous thrombolysis (38.4%), while fewer received adjunct therapy to MT, including intraarterial thrombolysis (7.8%), angioplasty (2.9%), and intracranial stenting (2.9%). The median procedure time for the entire cohort was 40 min (IQR 22–64 min) with a median number of 2 thrombectomy attempts (IQR 1–3 attempts).

The exact M2 occlusion location was reported in 66.7% of cases (*n* = 1154). The M2 trunk was most frequently the site of occlusion, representing 47.8% (*n* = 552) of these cases, with the remainder almost equally divided between the frontal (26.2%, *n* = 302) and temporal (26.0%, *n* = 300) divisions. Laterality was reported in 1234 cases, with a roughly 2:1 distribution of left versus right hemisphere M2 laterality (63.4%, *n* = 782% vs 36.6%, *n* = 452).

MT technique employed was frontline aspiration in 44.8% (711/1734) of procedures, while the remainder utilized stent retriever or combined techniques (55.2%, 958/1734). There were significant differences in baseline characteristics between the two technique groups for gender (*p* = 0.048), age (*p* < 0.001), race/ethnicity (*p* < 0.01), history of atrial fibrillation (*p* = 0.006), history of congestive heart failure (*p* = 0.003), prior stroke (*p* = 0.036), and smoking status (<0.001). There were no differences in stroke severity measured by NIHSS score (*p* = 0.265) or ASPECTS (*p* = 0.588). Frontline aspiration was less likely to be preceded by IV thrombolysis (34.0% vs 42.0%, *p* < 0.001). The overall distribution of the M2 occlusion site was significantly different for laterality (*p* = 0.008) and segment (*p* = 0.012) between groups, with frontline aspiration employed more often for left M2 (66.9% vs 59.5%) and M2 trunk (52.2% vs 43.5%) occlusions.

#### Outcomes before PSM

The overall primary outcome of functional independence rate at 90 days was 46.5% (Table 2). Successful and complete recanalization occurred in 88.5% and 53.4%, with modified first pass and complete first pass rates of 43.7% and 30.2%, respectively. Procedural complication rates were distal embolization (11.2%), any hemorrhage (21.2%), and sICH (4.7%). There was an improvement in NIHSS (median discharge score four, IQR 1–11) with a median reduction of five points (IQR −10.5– to −1.5) from admission to discharge. Secondary clinical outcomes at 3-month follow-up showed a median mRS of 2 (IQR 1–4) an excellent functional outcome rate (mRS 0–1) of 34.1%, and a mortality rate of 19.6%. The futile recanalization rate was 45.3%.

**Table 2.** Interventional outcomes.

Outcomes	Overall (*n* = 1734)	Frontline aspiration (*n* = 776)	SR/combined (*n* = 958)	Effect size**
Functional Independence at 90 days (mRS 0–2)*, *n* (%)	806 (46.5)	384 (49.5)	422 (44.1)	**1.24 (1.02–1.51)**
Successful recanalization (TICI 2B or better), *n* (%)	1534 (88.5)	692 (89.2)	842 (87.9)	1.14 (0.82–1.57)
Complete recanalization (TICI 2C or better), *n* (%)	926 (53.4)	472 (60.8)	454 (47.4)	**1.72 (1.42–2.09)**
Modified first pass effect, *n* (%)	758 (43.7)	352 (45.4)	406 (42.4)	1.13 (0.93–1.37)
Complete First pass effect, *n* (%)	524 (30.2)	267 (34.4)	257 (26.8)	**1.43 (1.16–1.76)**
Distal embolization, *n* (%)	195 (11.2)	94 (12.1)	101 (10.5)	1.17 (0.86–1.58)
Any hemorrhage, *n* (%)	368 (21.2)	174 (22.4)	194 (20.3)	1.14 (0.90–1.43)
Symptomatic hemorrhage, *n* (%)	82 (4.7)	27 (3.5)	55 (5.7)	**0.59 (0.37–0.95)**
90-day mRS, median [IQR]	2 [1–4]	2 [1–4]	3 [1–4]	**(−0.31) [(−0.61), (−0.11)]**
90-day mRS 0–1, *n* (%)	591 (34.1)	282 (36.3)	309 (32.3)	1.19 (0.98–1.46)
Discharge NIHSS, median [IQR]	4 [1–11]	4 [1–10]	5 [2–12]	**(−1.15) [(−1.91), (−0.39)]**
Change in NIHSS score from admission to discharge, median [IQR]	(−5) [(−10.5), (−1.5)]	(−5) [(−11), (−2)]	(−5.5) [(−10), (−1)]	**0.82 [0.04, 1.60]**
Improved NIHSS, *n* (%)	1401 (80.8)	614 (79.1)	787 (82.2)	0.83 (0.64–1.07)
Futile recanalization, *n* (%)	786 (45.3)	333 (42.9)	453 (47.3)	0.84 (0.69–1.01)
90-day mortality, *n* (%)	340 (19.6)	151 (19.5)	189 (19.7)	0.98 (0.77–1.25)

Overall and unmatched frontline thrombectomy cohorts.

^*^Primary outcome.

^**^Effect Size for comparison of frontline aspiration and SR/combined frontline thrombectomy techniques, values in bold indicate statistically significant differences.

Univariate subgroup analysis by MT technique showed a significant primary outcome benefit for frontline aspiration compared to stent retrievers or combined techniques with higher rates of functional independence at 90 days (49.5% vs 44.1%; OR 1.24, 95% CI 1.02–1.51). Secondary outcomes also favored frontline aspiration for complete recanalization (60.8% vs 47.4%; OR 1.72, 95% CI 1.42–2.09), complete first pass effect (34.4% vs 26.8%; OR 1.43, 95% CI 1.16–1.76), lower 90-day median mRS (2, IQR 1–4 vs 3, IQR 1–4; *p* = 0.04), and lower median NIHSS at discharge (4, IQR 1–10 vs 5, IQR 2–12; *p* = 0.003). There was a higher median NIHSS decrease at discharge from admission with SR/combined (−5, IQR −11 to –−2 vs −5.5, IQR −10 to −1; *p* = 0.04). frontline aspiration had significantly lower rates of sICH (3.5% vs 5.7%, *p* = 0.03). There were no differences in futile recanalization or mortality. (Table 2).

#### Outcomes after PSM

In the PSM analysis comparing frontline aspiration to SR/combined, cohorts (matched pairs, *n* = 711) were well-balanced for baseline demographics, time from symptom onset to arterial puncture, utilization of adjunct techniques and balloon guide catheters, and number of thrombectomy attempts (Table 3). The matching adequacy was assessed by comparing the distribution of PSs between pre- and post-matched datasets (Supplemental Figure 1). A notable significant difference between groups was shorter procedure time with frontline aspiration (28 min, IQR 15–49.5 min vs SR/combined 51 min, IQR 35–78 min; *p* < 0.001) after PSM. Analysis after PSM showed significantly higher rates functional independence at 90 days (49.9% vs 44.0%, OR 1.27, 95% CI 1.03–1.57), complete recanalization (61.2% vs 48.7%, OR 1.66, 95% CI 1.34–2.05), complete first pass effect (35.0% vs 27.6%, OR 1.42, 95% CI 1.13–1.78) in frontline aspiration compared to SR/combined (Table 4). After PSM, frontline aspiration had significantly lower rates of sICH (3.5% vs 6.2%, OR 0.55, 95% CI 0.33–0.91), lower median of NIHSS at discharge (4, IQR 1–9 vs 5, IQR 2–13, *p* = 0.04), and higher rates of improved NIHSS at discharge (83.4% vs 79.2%, OR 1.32, 95% CI 1.00–1.74) compared to stent retrievers or combined techniques. There were no differences in rates of excellent functional outcomes, mortality, or futile recanalization.

**Table 3.** Baseline demographics and procedural characteristics.

Baseline characteristics	CA (*n* = 711)	SR/combined (*n* = 711)	SMD	*p*-value
Sex, female, *n* (%)	353 (49.6)	357 (50.2)	0.01	0.874
Age, years, mean (SD)	69.6 (14.3)	70.3 (13.9)	0.05	0.376
Race or ethnicity, *n* (%)				0.823
White	494 (69.5)	500 (70.3)	0.02	
Black	119 (16.7)	115 (16.2)	0.01	
Hispanic	44 (6.2)	41 (5.8)	0.02	
Other	54 (7.6)	55 (7.7)	0	
Hypertension, *n* (%)	527 (74.1)	523 (73.6)	0.01	0.856
Diabetes, *n* (%)	224 (31.5)	231 (32.5)	0.02	0.733
Atrial fibrillation, *n* (%)	262 (36.8)	267 (37.6)	0.02	0.826
Hyperlipidemia, *n* (%)	344 (48.4)	344 (48.4)	0	1
Congestive heart failure, *n* (%)	112 (15.8)	102 (14.3)	0.04	0.505
Prior stroke, *n* (%)	125 (17.6)	120 (16.9)	0.02	0.779
Pre-stroke mRS, median [IQR]	0 [0–1]	0 [0–1]	0.03	0.588
Smoking, *n* (%)				0.185
Non smoker	492 (69.2)	525 (73.8)	0.1	
Prior smoker	120 (16.9)	90 (12.7)	0.12	
Current smoker	99 (13.9)	96 (13.5)	0.01	
Admission NIHSS, median [IQR]	12 [7–18]	12 [7–18]	0	0.689
ASPECTs, median [IQR]	8 [8–10]	8 [8–10]	0	0.431
Time from onset to arterial puncture, min, mean (SD)	444.5 (426.8)	459 (786.3)	0.02	0.648
Intravenous thrombolysis, *n* (%)	253 (35.6)	261 (36.7)	0.02	0.699
Intraarterial thrombolysis, *n* (%)	60 (8.4)	59 (8.3)	0.02	0.999
Angioplasty, *n* (%)	21 (3.0)	21 (3.0)	0	1
Intracranial stent, *n* (%)	16 (2.2)	15 (2.1)	0.01	0.999
Balloon guide catheter, *n* (%)	108 (15.2)	120 (16.9)	0.05	0.427

SMD: standardized mean difference.

Propensity score-matched cohorts comparing frontline thrombectomy technique. Cohorts balanced for all predictor variables except for procedure time.

**Table 4.** Interventional outcomes.

Outcomes	CA (*n* = 711)	SR/combined (*n* = 711)	Effect size**
Procedure time, min, median [IQR]	28 [15–49.5]	51 [35–78]	**0.85 [0.22–2.12]**
Number of attempts, median [IQR]	2 [1–3]	2 [1–3]	0
Functional independence at 90 days (mRS 0–2)*, *n* (%)	355 (49.9)	313 (44.0)	**1.27 [1.03–1.57]**
Successful recanalization (TICI 2B or better), *n* (%)	640 (90.0)	623 (87.6)	1.27 [0.90–1.79]
Complete recanalization (TICI 2C or better), *n* (%)	435 (61.2)	346 (48.7)	**1.66 [1.34–2.05]**
Modified first pass effect, *n* (%)	329 (46.3)	303 (42.6)	1.16 [0.94–1.43]
Complete first pass effect, *n* (%)	249 (35.0)	196 (27.6)	**1.42 [1.13–1.78]**
Distal embolization, *n* (%)	80 (11.3)	75 (10.5)	1.08 [0.77–1.51]
Any hemorrhage, *n* (%)	158 (22.2)	149 (21.0)	1.08 [0.84–1.39]
Symptomatic hemorrhage, *n* (%)	25 (3.5)	44 (6.2)	**0.55 [0.33–0.91]**
90-day mRS, median [IQR]	3 [1–5]	3 [1–5]	(−0.15) [(−0.52), (0.22)]
90-day mRS 0–1, *n* (%)	260 (36.6)	248 (34.9)	1.08 [0.86–1.34]
Discharge NIHSS, median [IQR]	4 [1–9]	5 [2–13]	**(−0.89) [(−1.74), (−0.04)]**
Change in NIHSS score from admission to discharge, median [IQR]	(−7) [(−12), (−3)]	(−7) [(−13), (−3)]	(0.21) [(−0.33), (0.75)]
Improved NIHSS, *n* (%)	593 (83.4)	563 (79.2)	**1.32 [1.00–1.74]**
Futile recanalization, *n* (%)	309 (43.5)	314 (44.2)	0.97 [0.79–1.20]
90-day mortality, *n* (%)	136 (19.1)	127 (17.9)	1.09 [0.83–1.42]

Efficacy and safety outcomes comparing propensity score matched cohorts.

^*^Primary outcome.

^**^Effect size for comparison of frontline aspiration and SR/combined frontline thrombectomy techniques, values in bold indicate statistically significant differences.

### Subgroup analysis of thrombectomy technique by M2 occlusion segment and laterality

**Figure 2.**
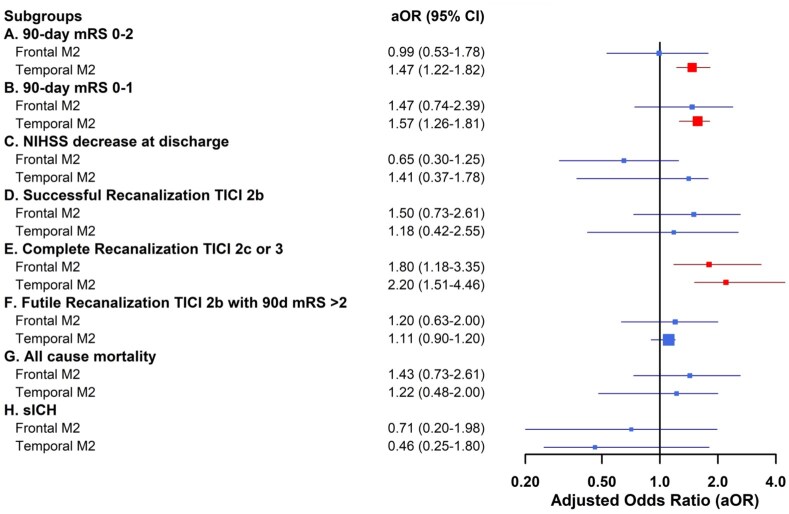
Subgroup Analysis of Recanalization, Safety, and Functional Outcomes of M2 occlusion segments (Frontal & Temporal) by Frontline Thrombectomy Technique. Forest plot shows adjusted odds ratios and 95% confidence intervals for (a) Functional independence (mRS 0–2) at 90 days, (b) Excellent functional outcome (mRS 0–1) at 90 days, (c) NIHSS, (d) Successful recanalization (TICI 2b or better), (e) Complete recanalization (TICI 2c or better), (f) Futile recanalization (TICI 2b or better with 90-day mRS > 2), decrease at discharge, (g) mortality, (h) symptomatic intracranial hemorrhage (sICH). Abscissa indicates direction of effect favoring frontline aspiration as indicated. Blue box indicates significant difference (*p* < 0.05), red indicates non-significance. See Supplemental Tables 1a–f for details.

**Figure 3.**
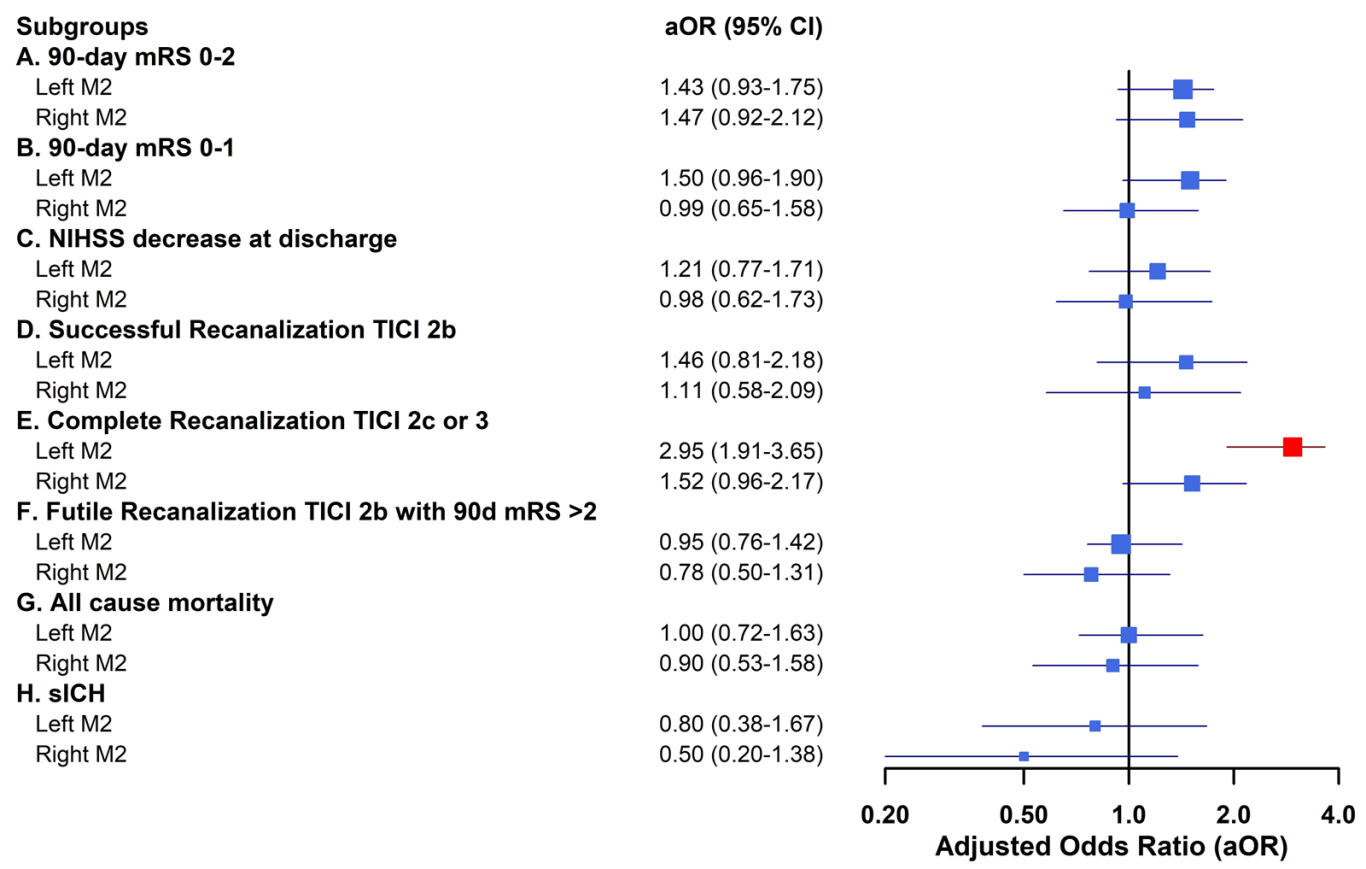
Subgroup Analysis of Recanalization, Safety, and Functional Outcomes of M2 Occlusion Laterality (Left & Right) by Frontline Thrombectomy Technique. Forest plot shows adjusted odds ratios and 95% confidence intervals for (a) Functional independence (mRS 0–2) at 90 days, (b) Excellent functional outcome (mRS 0–1) at 90 days, (c) NIHSS, (d) Successful recanalization (TICI 2b or better), (e) Complete recanalization (TICI 2c or better), (f) Futile recanalization (TICI 2b or better with 90-day mRS > 2), decrease at discharge, (g) mortality, (h) symptomatic intracranial hemorrhage (sICH). Abscissa indicates direction of effect favoring frontline aspiration as indicated. Blue box indicates significant difference (*p* < 0.05), red indicates non-significance. See Supplemental Tables 1a–f for details.

In patients with temporal M2 occlusions, contact aspiration was associated with significantly better functional outcomes. Specifically, these patients had higher odds of achieving functional independence at 90 days (mRS 0–2; aOR 1.47, 95% CI 1.22–1.82) and excellent functional outcomes (mRS 0–1; aOR 1.57, 95% CI 1.26–1.81). No significant functional benefit was observed for frontal M2 occlusions. For recanalization, contact aspiration was associated with higher odds of complete reperfusion (TICI 2c or three) in multiple subgroups, including frontal M2 occlusions (aOR 1.80, 95% CI 1.18–3.35), temporal M2 occlusions (aOR 2.20, 95% CI 1.51–4.46), and particularly left-sided M2 occlusions (aOR 2.95, 95% CI 1.91–3.65). Across all subgroups, there were no significant differences in safety outcomes, including all-cause mortality and symptomatic intracranial hemorrhage (sICH) between thrombectomy techniques (Figures 2 and 3).

### Impact of procedure time by technique

**Figure 4.**
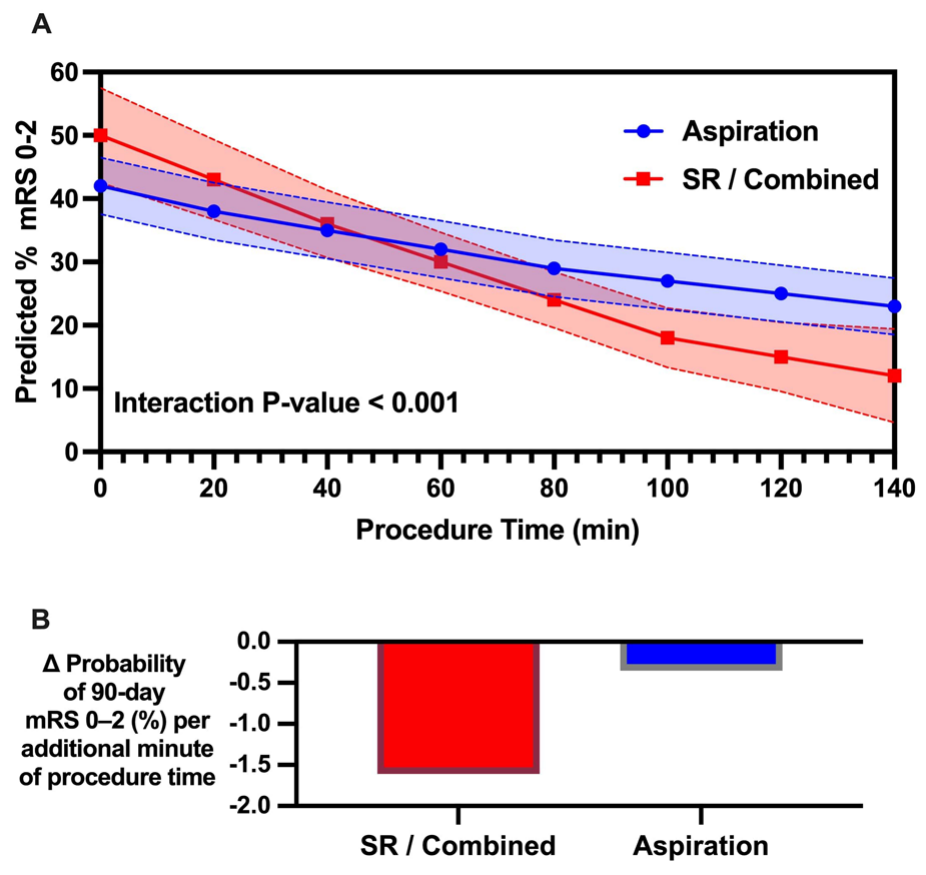
(a) Marginal effects of procedure time to successful recanalization on 90-day functional independence (mRS 0–2) in M2 occlusions by frontline technique. (b) Change in probability of functional independence per additional minute of procedure time by technique.

The impact of procedure time on outcomes was evaluated using marginal effects analysis (Figure 4). In the PSM cohort, frontline aspiration achieved successful recanalization a median of 23 min faster than SR/combined. There was a significant interaction between first-line technique and procedure time on the probability of achieving 90-day functional independence. The slope for aspiration was −0.0035, indicating a 0.35% decrease in mRS 0–2 probability per additional minute, compared to a slope of −0.0161 (1.61% decrease per minute) for SR/combined. The between-technique difference in slopes was −0.0126 (1.26% per minute; *p* < 0.001), favoring aspiration.

## Discussion

Compared with the selected populations of recent MeVO thrombectomy trials, our real-world registry captures a broader spectrum of patients with M2 occlusions, including those who are younger and present with a wider range of stroke severity. Within this setting, frontline contact aspiration was associated with better functional outcomes, more complete reperfusion, and preserved safety. These benefits were influenced by anatomical factors, with M2 segment and laterality modifying the magnitude of effect. Procedural time also emerged as a key driver of outcome. Frontline contact aspiration achieved faster reperfusion, and the negative impact of procedure time prolongation on functional outcomes was less pronounced with frontline aspiration than with stent retriever or combined approaches.

In 2025, three randomized trials, ESCAPE-MeVO, DISTAL, and DISCOUNT—found no superiority of MT over medical management for MeVO,^27,28^ fueling ongoing uncertainty despite prior non-level-1 evidence. The investigators in DISTAL excluded dominant-M2 segment MeVO, citing results from the HERMES collaboration M2 analysis that MT was beneficial for these occlusions. Despite this pragmatic enrollment approach, we are still left without a beachhead of level 1 evidence proving MT efficacy for any MeVO population.^29^

Within this landscape, M2 occlusions have consistently shown the greatest signal of MT benefit for MeVO in previous studies,^10,11^ with both Society of NeuroInterventional Surgery and American Heart Association/American Stroke Association guidelines endorsing endovascular treatment of M2 occlusions in reasonably selected patients.^7,30,31^ Published data on frontline thrombectomy technique for M2 occlusions show conflicting results. In 465 patients from Renieri et al., aspiration was associated with lower successful reperfusion (66.7% vs 84.2%) and functional independence (53.3% vs 62.2%) compared to stent retrievers.^32^ Conversely, Fifi et al., in a prospective global registry of 113 M2 occlusions, reported higher functional independence (83.1% vs 57.1%), greater successful reperfusion (85.3% vs 69.8%), and lower mortality (4.4% vs 16.3%) with aspiration. The ETIS registry, including 458 occlusions, found no significant difference in functional outcome or reperfusion between techniques but observed fewer procedural complications (aOR 0.39) and lower mortality (aOR 0.38) with aspiration.^33^ Saber et al., in 340 patients, reported no difference in 90-day functional independence between stent retriever and aspiration (57.6% vs 48.6%).^11^ A meta-analysis by Wen et al., pooling 601 patients from five observational studies published up to 2021, found comparable rates of final successful reperfusion (OR 1.18, 95% CI 0.72–1.93) and 90-day functional independence (OR 1.18, 95% CI 0.82–1.68) between first-line aspiration and stent retriever.^34^ Our study revisits this question using 1734 STAR registry patients—more than all studies in that meta-analysis combined—treated through 2024, reflecting modern device technology and an increased ability to tailor catheters and devices to specific M2 anatomies. In this large real-world U.S. cohort, contact aspiration was superior for complete reperfusion and functional independence, with the magnitude of benefit modified by M2 segment, laterality, and the interaction between technique and procedure time. These findings reinforce that the benefit of mechanical thrombectomy in M2 occlusions is strongly shaped by both technical and anatomical factors, and future randomized trials should incorporate these considerations when designing patient selection criteria and procedural strategies.

Hemispheric involvement emerged as a meaningful determinant of outcome, with left-sided occlusions demonstrating greater benefit from mechanical thrombectomy, particularly when treated with frontline aspiration. This effect was most pronounced in temporal M2 occlusions, where both technical and functional considerations likely converge. The temporal division is frequently dominant in flow, with a wider lumen and more favorable take-off from the M1 trunk, features that facilitate rapid and secure catheter engagement and optimize aspiration seal. The laterality signal may similarly be explained by cortical eloquence. Left hemisphere strokes more often compromise language and dominant-hand motor areas, making neurological outcomes acutely sensitive to the timeliness and quality of reperfusion.^12,19,35^

Procedure time further modified outcomes in a technique-dependent manner. With stent retriever or combined approaches, each additional minute was associated with a sharper decline in the probability of functional independence, whereas this penalty was notably attenuated with aspiration. The mechanistic basis is intuitive: aspiration consistently shortens access-to-recanalization intervals as indicated in our cohort, reducing the duration of ischemia, and may better contain clot fragments, preserving distal microcirculation. Previous reports have shown that aspiration is more time-sensitive, while stent retriever outcomes are more strongly influenced by the number of attempts. In our cohort, the procedural time advantage conferred by aspiration devices was particularly pronounced and outperformed stent retriever approaches when restricted to M2 occlusions. Even when aspiration cases run longer, it seems that the less mechanical manipulation may limit endothelial trauma and secondary ischemic injury compared to stent retriever deployment.

Taken together, these findings suggest that in real-world M2 occlusion thrombectomy, aspiration offers a dual advantage, faster reperfusion and reduced susceptibility to the adverse effects of procedural delay. Certain anatomic subtypes, particularly temporal M2 and left-sided occlusions, appear to derive disproportionate benefit, underscoring the need for an individualized approach to device selection. Future trials should move beyond treating “MeVO” as a monolith, incorporating standardized anatomic definitions, segment-level and laterality stratification, and procedural efficiency metrics to identify the subpopulations most likely to benefit from each thrombectomy technique.

### Limitations

This study has several important limitations. First, as a retrospective multicenter registry analysis, it is inherently subject to selection bias, particularly since first-line technique choice is operator-dependent. Second, our inclusion of all M2 occlusions provides a broad real-world view but also incorporates M2 trunk occlusions—often classified and treated as LVOs in other studies (e.g. MOSTE, NCT03796468)—and cases without a clearly defined segment. Excluding these and focusing solely on distal or dominant M2 occlusions would better align with the DMVO populations targeted by ongoing trials, but detailed data on proximal versus distal involvement and MCA dominance were not available. Finally, the M2 segment represents a transitional zone between large and medium vessel occlusions, and the absence of systematic classification by segment location and dominance may dilute interpretive clarity. Third, outcomes were not core lab–adjudicated, introducing potential variability across centers, though we adjusted for site-level effects in our models. Future studies with detailed angiographic classification and standardized inclusion criteria are needed to refine patient selection and enhance replicability.

## Conclusion

In the largest series to date (*n* = 1734) from a multicenter U.S. stroke registry, patients with acute MCA M2 occlusions treated with mechanical thrombectomy achieved higher rates of functional independence at 90 days when frontline aspiration was used compared to stent retriever or combined approaches. These results contrast with recent MeVO trials, dominated by stent retriever strategies, that failed to show benefit over medical therapy, and highlight that technique selection, vascular anatomy, and procedural efficiency can critically influence outcomes. In current practice, these findings support greater consideration of aspiration as a first-line approach for M2 occlusions, particularly in anatomically favorable segments. Future randomized trials should stratify by M2 anatomy, laterality, and procedural time to identify the MeVO subgroups most likely to benefit from thrombectomy and to establish level 1 evidence for optimal device selection.

## Supplementary Material

ds-eso_23969873251381924
