# Supplementary material for: Frontline aspiration versus stent retriever thrombectomy for M2 occlusions: Insights from the STAR registry
Source: Eur Stroke J. 2026 Jan 1;11(1):23969873251381924. doi: 10.1093/esj/23969873251381924 (PMC12866269; doi:10.1093/esj/23969873251381924)
Supplement: ds-eso_23969873251381924 [file ds-eso_23969873251381924.zip › sj-docx-1-eso-10.1177_23969873251381924.docx]

**Supplemental Content**

1. Supplemental Tables 1
2. Supplemental Figures 1

| **Location** | **aOR** | **Lower CI** | **Upper CI** |
| --- | --- | --- | --- |
| Left M2 | 1.43 | 0.93 | 1.75 |
| Right M2 | 1.47 | 0.92 | 2.12 |
| Frontal M2 | 0.99 | 0.53 | 1.78 |
| Left Frontal M2 | 0.96 | 0.44 | 2.32 |
| Right Frontal M2 | 0.97 | 0.47 | 1.49 |
| Temporal M2 | **1.47** | **1.22** | **1.82** |
| Left Temporal M2 | **1.5** | **1.23** | **2.09** |
| Right Temporal M2 | 1.44 | 0.99 | 2.17 |

**Table 1a. Functional independence at** **90-days (mRS 0-2).** Adjusted subgroup analysis by frontline technique for MCA laterality and segment. (aOR)>1 favors ADAPT. Benefit for ADAPT in Temporal M2 and Left Temporal M2 occlusions, no other differences seen.

| **Location** | **aOR** | **Lower CI** | **Upper CI** |
| --- | --- | --- | --- |
| Left M2 | 1.5 | 0.96 | 1.9 |
| Right M2 | 0.99 | 0.65 | 1.58 |
| Frontal M2 | 1.47 | 0.74 | 2.39 |
| Left Frontal M2 | 1.5 | 0.58 | 2.86 |
| Right Frontal M2 | 1.49 | 0.72 | 2 |
| Temporal M2 | **1.57** | **1.26** | **1.81** |
| Left Temporal M2 | **1.52** | **1.19** | **2** |
| Right Temporal M2 | 1.49 | 0.98 | 2 |

**Table 1b. Excellent Functional Outcome at** **90-days (mRS 0-1).** Adjusted subgroup analysis by MCA laterality and segment. (aOR)>1 favors ADAPT. Benefit for ADAPT in Temporal M2 and Left Temporal M2 occlusions, no other differences seen.

| **Location** | **aOR** | **Lower CI** | **Upper CI** |
| --- | --- | --- | --- |
| Left M2 | 1.21 | 0.77 | 1.71 |
| Right M2 | 0.98 | 0.62 | 1.73 |
| Frontal M2 | 0.65 | 0.3 | 1.25 |
| Left Frontal M2 | **0.33** | **0.19** | **0.91** |
| Right Frontal M2 | **0.35** | **0.19** | **0.62** |
| Temporal M2 | 1.41 | 0.37 | 1.78 |
| Left Temporal M2 | 1.4 | 0.8 | 1.95 |
| Right Temporal M2 | 1.1 | 0.53 | 1.97 |

**Table 1c.** **NIHSS decrease at discharge.** Adjusted subgroup analysis by MCA laterality and segment. (aOR)>1 favors ADAPT. Benefit for ADAPT in Left Frontal M2 and Right Frontal M2 occlusions, no other differences seen.

| **Location** | **aOR** | **Lower CI** | **Upper CI** |
| --- | --- | --- | --- |
| Left M2 | 1.46 | 0.81 | 2.18 |
| Right M2 | 1.11 | 0.58 | 2.09 |
| Frontal M2 | 1.5 | 0.73 | 2.61 |
| Left Frontal M2 | 1.08 | 0.3 | 2.61 |
| Right Frontal M2 | 1.24 | 0.25 | 2.62 |
| Temporal M2 | 1.18 | 0.42 | 2.55 |
| Left Temporal M2 | 1.02 | 0.35 | 2.24 |
| Right Temporal M2 | 1.24 | 0.18 | 2.36 |

**Table 1d.** **Successful Recanalization (TICI 2b or better).** Adjusted subgroup analysis by MCA laterality and segment. (aOR)>1 favors ADAPT. No differences seen.

|  |  |  |  |
| --- | --- | --- | --- |
| **Location** | **aOR** | **Lower CI** | **Upper CI** |
| Left M2 | **2.95** | **1.91** | **3.65** |
| Right M2 | 1.52 | 0.96 | 2.17 |
| Frontal M2 | **1.8** | **1.18** | **3.35** |
| Left Frontal M2 | **3.8** | **1.76** | **7.81** |
| Right Frontal M2 | **1.98** | **1.38** | **3.65** |
| Temporal M2 | **2.2** | **1.51** | **4.46** |
| Left Temporal M2 | **3.51** | **1.4** | **4.67** |
| Right Temporal M2 | **1.9** | **1.01** | **4.98** |

**Table 1e.** **Complete Recanalization (TICI 2c or 3).** Adjusted subgroup analysis by MCA laterality and segment. (aOR)>1 favors ADAPT. Benefit for ADAPT in all cases except for no difference for Right M2 occlusions.

|  |  |  |  |
| --- | --- | --- | --- |
| **Location** | **aOR** | **Lower CI** | **Upper CI** |
| Left M2 | 0.95 | 0.76 | 1.42 |
| Right M2 | 0.78 | 0.5 | 1.31 |
| Frontal M2 | 1.2 | 0.63 | 2 |
| Left Frontal M2 | 0.99 | 0.43 | 2.33 |
| Right Frontal M2 | 1.23 | 1.2 | 1.24 |
| Temporal M2 | **1.11** | **0.9** | **1.2** |
| Left Temporal M2 | 0.87 | 0.76 | 1.52 |
| Right Temporal M2 | 0.99 | 0.55 | 1.48 |

**Table 1f.** **Futile Recanalization TICI 2b with 90d mRS >2.** Adjusted subgroup analysis by MCA laterality and segment. (aOR)<1 favors ADAPT. Benefit for SR/combined approaches in Temporal M2 occlusion, no other differences seen.

| **Location** | **aOR** | **Lower CI** | **Upper CI** |
| --- | --- | --- | --- |
| Left M2 | 1 | 0.72 | 1.63 |
| Right M2 | 0.9 | 0.53 | 1.58 |
| Frontal M2 | 1.43 | 0.73 | 2.61 |
| Left Frontal M2 | 1.72 | 0.71 | 2.61 |
| Right Frontal M2 | 1.64 | 0.56 | 2.62 |
| Temporal M2 | 1.22 | 0.48 | 2 |
| Left Temporal M2 | 1.64 | 0.55 | 1.81 |
| Right Temporal M2 | 0.71 | 0.45 | 1.35 |

**Table 1g.** **All cause mortality at 90 days.** Adjusted subgroup analysis by MCA laterality and segment. (aOR)<1 favors ADAPT. No differences seen.

|  |  |  |  |
| --- | --- | --- | --- |
| **Location** | **aOR** | **Lower CI** | **Upper CI** |
| Left M2 | 0.8 | 0.38 | 1.67 |
| Right M2 | 0.5 | 0.2 | 1.38 |
| Frontal M2 | 0.71 | 0.2 | 1.98 |
| Left Frontal M2 | 0.46 | 0.17 | 2.25 |
| Right Frontal M2 | 0.95 | 0.47 | 1.61 |
| Temporal M2 | 0.46 | 0.25 | 1.8 |
| Left Temporal M2 | 0.83 | 0.47 | 1.7 |
| Right Temporal M2 | 0.4 | 0.19 | 1.17 |

**Table 1h. Symptomatic Intracranial Hemorrhage.** Adjusted subgroup analysis by MCA laterality and segment. (aOR)<1 favors ADAPT. No differences seen.


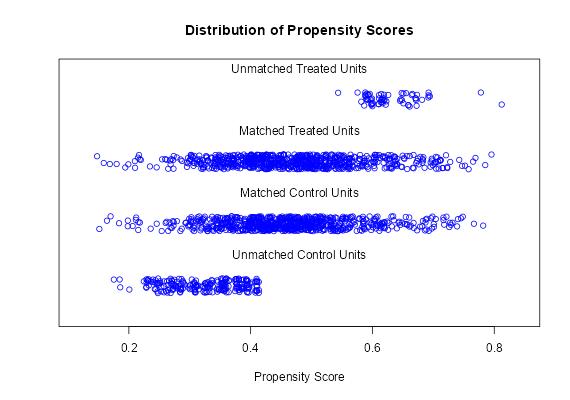


**Figure 1. Distribution of propensity scores for thrombectomy technique (ADAPT vs SR/combined) between pre- and post-matched datasets.** PSM was utilized to balance baseline characteristics between patients with ADAPT and those with SR/combined. PS matching was performed using major clinical variables showing standardized mean difference (SMD) >0.2 in univariate analysis. The matching algorithm employed was 1:1 nearest neighbor greedy matching without replacement optimal caliper width was determined to be 0.1 times the standard deviation of the logit of the PSs of all patients.
